# Case Report: Combined PD-1 and tyrosine kinase blockade stabilizes refractory pancreatic cancer guided by the spatial structure of tumor immune microenvironment

**DOI:** 10.3389/fimmu.2025.1547388

**Published:** 2025-05-01

**Authors:** Heqi Yang, Yuhang Ma, Chenyan Zhang, Qingqing Leng, Ke Cheng, Chengjian Zhao, Dan Cao

**Affiliations:** ^1^ Department of Medical Oncology, Cancer Center, West China Hospital, Sichuan University, Chengdu, Sichuan, China; ^2^ Division of Abdominal Tumor Multimodality Treatment, Cancer Center, West China Hospital, Sichuan University, Chengdu, Sichuan, China; ^3^ State Key Laboratory of Biotherapy and Cancer Center, West China Hospital, Sichuan University, and Collaborative Innovation Center for Biotherapy, Chengdu, Sichuan, China

**Keywords:** pancreatic cancer, tumor immune microenvironment, spatial structure, immunotherapy, targeted therapy

## Abstract

Pancreatic cancer is characterized by a poor prognosis and limited responsiveness to conventional therapies, presenting a substantial therapeutic challenge. Although chemotherapy remains the cornerstone of systemic treatment, options become scarce once frontline therapies fail. While targeted therapies and immunotherapies have emerged as potential alternatives, their efficacy in pancreatic cancer is not well established. As research advances, exploring the tumor immune microenvironment (TiME) of pancreatic cancer is crucial and holds significant potential for developing novel treatment strategies.We report a case of a pancreatic cancer patient who, after the failure of frontline and second-line treatments, was treated with a pioneering combination of targeted therapy and immunotherapy to modulate the unique TiME. The targeted agent, surufatinib, is a tyrosine kinase inhibitor (TKI) that targets vascular endothelial growth factor receptor (VEGFR) 1–3, fibroblast growth factor receptor 1 (FGFR1), and colony-stimulating factor 1 receptor (CSF-1R). The immunotherapy agent, toripalimab, is an immune checkpoint inhibitor targeting programmed cell death protein 1 (PD-1). Remarkably, the patient benefitted from this regimen, exhibiting stable disease, improved clinical symptoms, and prolonged progression-free survival. This case highlights the potential of personalized therapy in treating pancreatic cancer, particularly in patients with distinctive features of the TiME that may predict favorable responses to immunotherapy. Personalized strategies that consider the spatial structure and composition of the TiME may offer a promising avenue for achieving long-term progression-free survival in patients with pancreatic cancer.

## 1 Introduction

Pancreatic cancer (PC) remains one of the most challenging malignancies in oncology and is characterized by its aggressive behavior, late-stage diagnosis, and limited responsiveness to conventional therapies (1). Standard first-line treatments, typically involving chemotherapy, often yield only modest improvements in survival and are frequently accompanied by significant toxicity (2). Although some patients initially derive significant clinical benefits, therapeutic resistance ultimately develops. Consequently, there is an urgent need for more effective and personalized treatment strategies.

Recent breakthroughs in the field of immunotherapy have revolutionized the treatment landscape for various cancers, offering hope for better outcomes. However, the role of immunotherapy in PC, particularly as a subsequent line of treatment following the failure of standard regimens, is still evolving. With more in-depth research into the TiME, the unique characteristics of PC have become increasingly associated with potential responsiveness to immunotherapy (3).

Owing to the TiME of PC, monotherapy with immune checkpoint blockade (ICB) has shown limited efficacy. However, combination therapies hold great promise in enhancing the immune response for better therapeutic outcomes. In this study, we introduced a TKI combined with ICB as a therapeutic regimen depending on the patient’s specific TiME. The pretreatment TiME was characterized by extensive fibrous deposition, enrichment of inflammatory cells, and the presence of tertiary lymphoid structures (TLSs) formed by the recruitment of immune cells to the tumor frontier edge, indicating potential responsiveness to immunotherapy. TKIs inhibit the growth and spread of tumor cells by blocking the activity of specific tyrosine kinases. TKIs have the potential to modulate the TiME in the following ways: inhibition of angiogenesis, modulation of immune cell function, reduction in inflammation, and reduction in fibrogenesis (4). Although TKIs are not the standard treatment for PC, owing to their potential to reverse the immunosuppressive microenvironment, we have attempted to reverse immune resistance with a pioneering regimen combining TKIs with immune checkpoint inhibitors.

In the present case, we report the clinical course of a PC patient who, after failing to respond to first-line chemotherapy, exhibited remarkable progression-free survival to the novel treatment regimen on the basis of her TiME, which demonstrated an inimitable spatial structure, high programmed death-ligand 1 (PD-L1) expression, and abundant tertiary lymph nodes.

## 2 Case description

**Figure 1:**
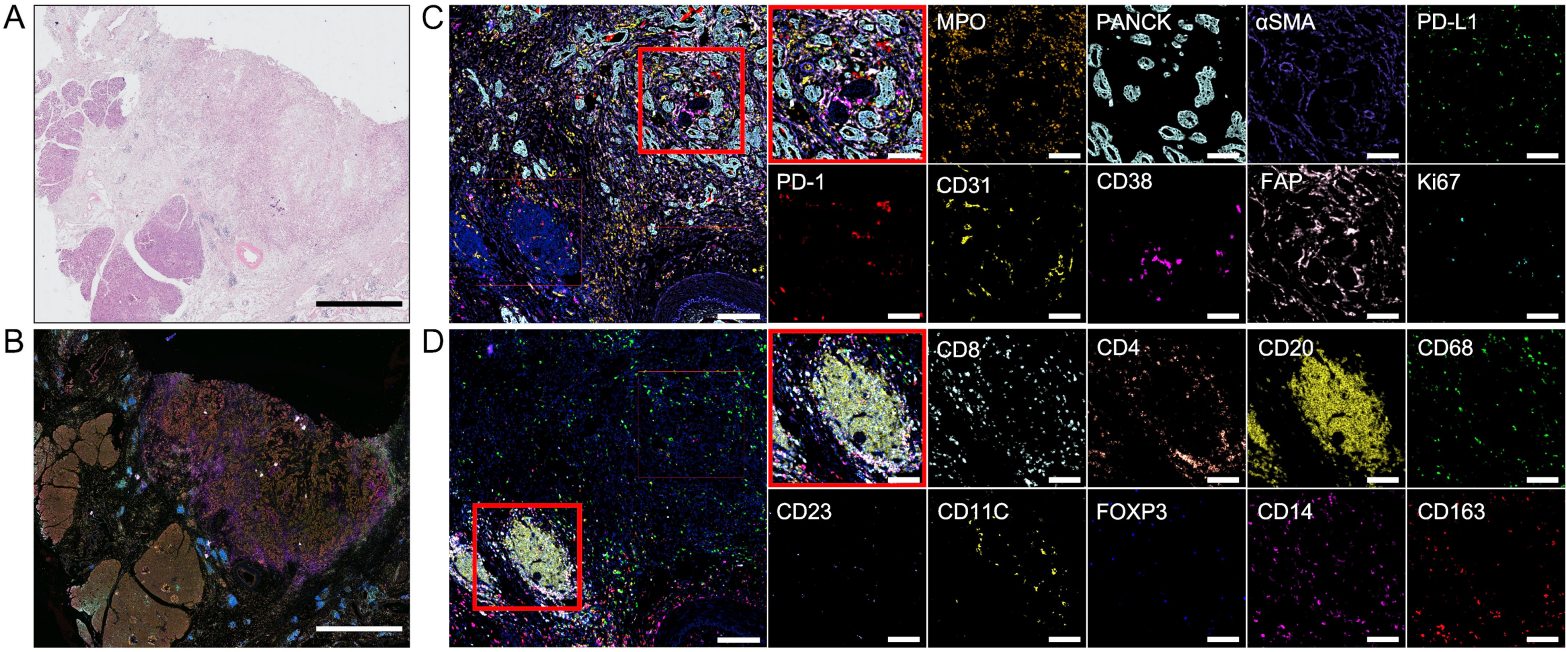
Hematoxylin and eosin (H&E) staining and multiplex immunohistochemistry. **(A)** Surgical biopsies stained with H&E. Scale bar: 5 mm. **(B)** Image of multiplex immunohistochemistry with 18 markers. Scale bar: 5 mm. **(C, D)** Images of one region of interest(ROI). The red box is used to select two smaller ROIs and show the different markers in them. ROI scale bar: 200 μm. Smaller ROI scale bar: 100 μm.

A patient in her early 60s presented with abdominal discomfort, accompanied by hunger and a burning sensation that improved after eating. She sought medical consultation at our hospital. After evaluation by a multidisciplinary team, the patient opted to undergo extended pancreaticoduodenectomy and pursue adjuvant therapy. Hematoxylin and eosin (H&E) staining (**Figure 1A**) and multiplex immunohistochemistry (mIHC) (**Figure 1B**) were performed on surgical biopsies from this patient. We employed the Cyclic-multiplex Tyramide Signal Amplification (CmTSA) platform for mIHC (5). This platform utilizes Tyramide Signal Amplification (TSA) combined with an efficient fluorophore recycling method, allowing for the labeling of 30–60 antigens across multiple tissue slides. The staining process was performed using conventional immunohistochemistry equipment and standard IHC primary antibodies, significantly reducing the overall costs. H&E staining confirmed that the pathological diagnosis was pancreatic ductal adenocarcinoma (PDAC). The TiME exhibited representative features of the immune microenvironment in PDAC. Notably, there was a particularly prominent mesenchymal compartment within the stroma, comprising the majority of the tumor volume (**Figure 1C**). This stromal compartment includes fibroblasts, extracellular matrix components, immune cells, and endothelial cells. Within the fibrous envelope, there was substantial expression of PD-1 and PD-L1. Additionally, the margins of the tumor tissues contained a significant number of TLSs, which are generally associated with a favorable prognosis (**Figure 1D**). The center of each TLS consists of large clusters of B cells surrounded by helper T cells that activate and enhance the immune response, aiding in antibody production.

**Figure 2:**
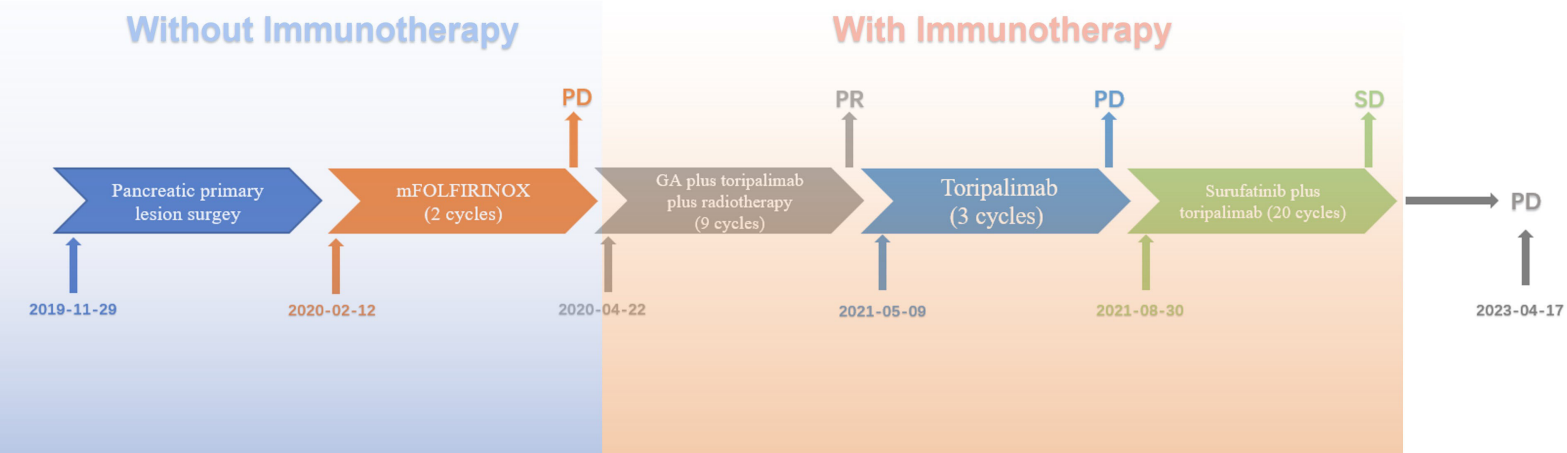
Schematic diagram of the antitumor treatment process.

Postoperatively, she began receiving systematic adjuvant therapy (**Figure 2**). She initiated frontline modified FOLFIRINOX (mFOLFIRINOX) regimen (oxaliplatin [85 mg per square meter of body surface area on day 1], irinotecan [150 mg per square meter on day 1], leucovorin [400 mg per square meter on day 1], and fluorouracil [2400 mg per square meter over a period of 46 hours] every 2 weeks). Over the next 2 cycles, the patient experienced progressive disease according to the Response Evaluation Criteria in Solid Tumors (RECIST) V.1.1. mFOLFIRINOX was discontinued, and the treatment strategy was revised to include combined chemotherapy, immunotherapy, and local radiation therapy. The patient received GA chemotherapy, which was composed of gemcitabine (1000 mg per square meter on days 1 and 8 every 3 weeks) and albumin-bound paclitaxel (125 mg per square meter on days 1 and 8 every 3 weeks) plus toripalimab (240 mg on day 1 every 3 weeks). The patient reported experiencing nausea, vomiting, and numbness in the hands and feet after the completion of treatment. The combined regimen was administered for 9 cycles, ultimately resulting in a partial response. On the basis of the favorable response, an adequate number of second-line treatment cycles, and the patient’s overall condition, we considered maintenance therapy with single-agent immunotherapy. She received only toripalimab (240 mg intravenously every 3 weeks) for 3 cycles. After completing the third cycle of treatment, a follow-up CT scan indicated baseline disease progression, along with an elevation in CA19-9 levels (S1A). The patient reported worsening abdominal pain and sought further treatment at an outside hospital where was treated with radioactive ^125^I seed implantation. To formulate the next treatment plan, we reviewed the mIHC images of the patient’s surgical specimens and the patient’s genetic test results to determine the potential for immunotherapy. There are no clear guidelines regarding whether PC patients should continue chemotherapy after failure of first- and second-line treatment regimens. Based on the patient’s overall condition, we discontinued chemotherapy and initiated targeted therapy combined with immunotherapy comprising surufatinib (250 mg every day) and toripalimab (240 mg intravenously every 3 weeks) in the 22nd month after the operation. Over the next 20 months, the patient experienced stable disease (S1B). When the patient was reexamined after completing 20 cycles, CT suggested tumor progression and ascites, and the patient presented with symptoms of hepatic encephalopathy, so treatment was discontinued.

## 3 Discussion

Despite advances in cancer treatment, the prognosis for PC patients, especially those with advanced stages, remains dismal. In addition to the inherent immune-evasive properties of PC, its highly immunosuppressive microenvironment further hinders the effectiveness of immunotherapy and targeted therapy. Consequently, the primary treatment for PC involves chemotherapy, which has a low response rate of less than 10% and a 5-year survival rate of 5–10% (1). Even with surgical intervention, 70% of early-stage patients progress to metastatic disease within a year, and recent studies have demonstrated minimal benefits from immunotherapy, whether used alone or in combination with targeted therapies (2).

**Figure 3:**
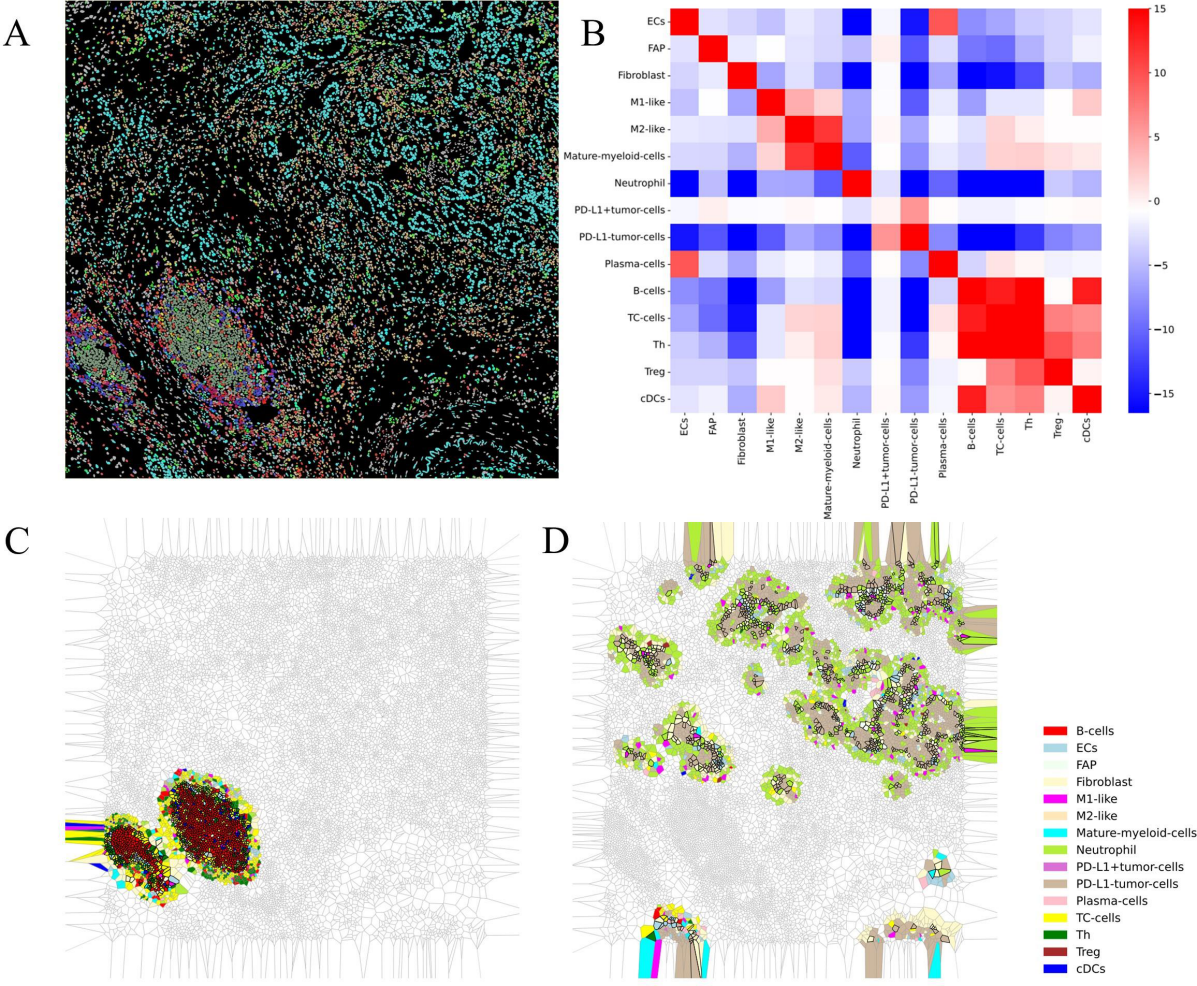
Results of vectorization and downstream analysis of multiplex immunohistochemistry images. **(A)** Space mapping of multiplex immunohistochemistry images generated from vectorized data. **(B)** Heatmap plot of the interaction analysis results. **(C, D)** Voronoi diagrams of spatial structures detected via downstream analysis.

In this case, the patient received the first-line mFOLFIRINOX regimen postsurgery and experienced local recurrence after just two cycles. Upon evaluating the patient’s baseline condition, we found that she exhibited positive PD-L1 expression, with a high tumor proportion score (TPS) of 30% and a combined positive score (CPS) of 32. However, according to previous reports, PD-L1 expression in patients with PDAC is not significantly correlated with the therapeutic efficacy of PD-1 inhibitors. To determine further treatment regimens, we considered the characteristics of the tumor microenvironment from the patient’s pretreatment biopsies. We selected a region of interest (ROI) from the biopsies with CmTSA staining (**Figure 1C**). The selected ROI was then processed using deep learning-based algorithms for accurate cell segmentation (5). Each cell within the ROI was annotated based on expression markers, allowing for the identification of different cell types. We identified 15 cell types with 18 markers. The ROI included a scattered arrangement of cancer cell nests and a tumor frontier region containing TLSs. After image vectorization (**Figure 3A**) and excluding unidentifiable cells, we counted 15,688 cells. Fibroblast activation proteins (FAPs) accounted for 1.8%, fibroblasts for 9.8%, and tumor cells for 12% of the ROI. The numbers of fibroblasts and tumor cells were similar, consistent with the characteristics of the PDAC TiME. In the tumor region (**Figure 1C**), we observed spatial proximity between PD-L1-expressing tumor cells and PD-1-expressing cells, suggesting the potential feasibility of anti-PD-1 immunotherapy. Consequently, the treatment was switched to a combined GA regimen with toripalimab alongside concurrent local radiotherapy, based on prior clinical trials that yielded positive results when pembrolizumab was combined with the GA regimen. The patient responded positively to this regimen, achieving a partial response at one point. However, local recurrence occurred again after maintenance monotherapy. These findings suggest that although many patients with PC have good immunotherapeutic potential, the immunosuppressive components of the microenvironment and physical isolation by fibrous structures present unique challenges to the efficacy of immune checkpoint inhibitors in this population. Therefore, determining the appropriate treatment on the basis of the specific tumor microenvironment is essential for personalized therapy. For example, treatment with gemcitabine and a CD40 agonist improves outcomes in patients with a low neutrophil-to-lymphocyte ratio (6). Additionally, patients may receive tailored regimens according to the different compositions of the tumor microenvironment, such as enrichment of FAP or collagen, enrichment of T cells or myeloid cells, or a poor immune cell profile (7).

According to previous reports, the TiME of PC is highly disorganized. It is typically characterized by dense stroma and immunosuppressive metabolic conditions, such as elevated lactate levels and hypoxia (8, 9). This tumor-specific environment not only promotes cancer growth and metastasis but also forms an immunosuppressive barrier that impedes both conventional and emerging immunotherapeutic strategies. Key features of the PC TiME include significant immune cell suppression, exemplified by the accumulation of regulatory T cells (Tregs) and myeloid-derived suppressor cells (MDSCs), as well as the presence of tumor-associated macrophages (TAMs). Additionally, the microenvironment contains a dense fibrotic matrix that physically hinders the infiltration of effective immune cells and chemically suppresses immune responses through the secretion of various immunosuppressive molecules (8).

To further determine the treatment plan, we analyzed the image vectorization data to identify the spatial distribution of these cell types, examining the interactions between paired cells and their clustering into functional multicellular niches via downstream analysis software (5). Interaction analysis revealed strong immune interactions among immune cells in the region, including antigen presentation and the activation of B cells and cytotoxic T cells (**Figure 3B**). Spatial structure prediction successfully identified TLSs (**Figure 3C**). Furthermore, we discovered that tumor cells, neutrophils, and fibroblasts form a unique structure (**Figure 3D**). In this configuration, neutrophils and cancer-associated fibroblasts (CAFs) surround tumor cells and create a dense stromal barrier through extracellular matrix (ECM) components, such as collagen and fibronectin, which are secreted by CAFs. These barriers not only physically restrict immune cell infiltration but also directly inhibit immune cell function via matrix components (10). This structure may contribute to an immunosuppressive microenvironment due to the presence of immunosuppressive factors such as TGF-β, IL-10, and VEGF, which are secreted by neutrophils and CAFs. These factors can inhibit the function of effector T cells and promote the expansion of Tregs, thereby further enhancing the immunosuppressive state (11–13). Given the tumor microenvironment and the patient’s baseline condition, chemotherapy was abandoned in favor of combination therapy consisting of a TKI, which can reduce inflammation and fibrogenesis, and PD-1 blockade. PD-1 blockade was selected based on the patient’s positive PD-L1 expression and the spatial proximity between PD-L1-expressing tumor cells and PD-1-expressing immune cells. The results from a phase II clinical trial using surufatinib combined with toripalimab in patients with advanced solid tumors indicate that this targeted and immunotherapy combination is well tolerated and demonstrates preliminary antitumor activity in these patients. Consequently, we considered treating the patient with a combination of surufatinib and toripalimab. Encouragingly, the patient achieved long-term progression-free survival of 20 months from combination therapy without unexpected safety signals; imaging and tumor marker assessments revealed tumor stabilization, resulting in stable disease.

Such prolonged progression-free survival is very rare in patients with recurrent PC after surgery. The pretreatment tumor microenvironment is characterized by extensive fibrous deposition, enrichment of inflammatory cells, and the presence of TLSs formed by the recruitment of immune cells to the frontier edge of the tumor, indicating the potential for immunotherapy. In accordance with these findings, the aim of adding TKIs is to modulate the microenvironment. Surufatinib significantly reduces tumor angiogenesis by inhibiting VEGFR and FGFR. A reduction in abnormal blood vessels and normalization of the vasculature improve the oxygen and nutrient supply in the tumor microenvironment, enabling immune cells to infiltrate the tumor more effectively (14, 15). By inhibiting CSF-1R, surufatinib decreases the number of TAMs and MDSCs, which, in collaboration with neutrophils and CAFs, enhance immunosuppressive effects (16). Additionally, surufatinib inhibits the activity and secretory functions of CAFs by targeting FGFR2, reducing the accumulation of the ECM, decreasing the rigidity of the tumor microenvironment, and increasing immune cell permeability. This allows effector T cells to more easily penetrate the tumor core for effective attack (12).

On the basis of existing clinical research and the patient’s unique tumor microenvironment structure, the potential mechanism for patient benefit may lie in the selection of appropriate targeted drugs combined with immunotherapy to alter the microenvironmental state (17, 18). Spatial analysis of the pretreatment tumor microenvironment revealed the spatial distribution of cells and the unique structures formed by these cells. Previous studies have indicated that the spatial organization of TiME plays a crucial role in predicting the efficacy of ICB, as multicellular spatial interactions significantly influence the treatment respons (19, 20). Exploring targeted therapies on the basis of the cellular composition of these structures is a future direction for precision treatment of PC. Unfortunately, we do not process on-treatment biopsies to determine the characteristics of the tumor microenvironment, making it impossible to accurately determine whether the spatial structure of the tumor microenvironment has changed, as we hypothesized under the combined treatment regimen.

## 4 Conclusion

In this case, the characteristics of the tumor immune microenvironment, particularly its unique spatial structure, guided the use of toripalimab alone and in combination with chemotherapy or surufatinib for refractory PC, which contributed to improve in survival outcomes. Future large-scale cohort studies are warranted to validate the efficacy and safe based the individual treatment strategy.

## Data Availability

The original contributions presented in the study are included in the article/**Supplementary Material**. Further inquiries can be directed to the corresponding authors.

## References

[1] Siegel RL Miller KD Fuchs HE Jemal A. Cancer statistics, 2022. CA: Cancer J Clin. (2022) 72:7–33. doi: 10.3322/caac.21708 35020204

[2] Wood LD Canto MI Jaffee EM Simeone DM. Pancreatic cancer: pathogenesis, screening, diagnosis, and treatment. Gastroenterology. (2022) 163:386–402.e1. doi: 10.1053/j.gastro.2022.03.056 35398344 PMC9516440

[3] Beatty GL Werba G Lyssiotis CA Simeone DM. The biological underpinnings of therapeutic resistance in pancreatic cancer. Genes Dev. (2021) 35:940–62. doi: 10.1101/gad.348523.121 PMC8247606 34117095

[4] Ebrahimi N Fardi E Ghaderi H Palizdar S Khorram R Vafadar R. Receptor tyrosine kinase inhibitors in cancer. Cell Mol Life sciences: CMLS. (2023) 80:104. doi: 10.1007/s00018-023-04729-4 36947256 PMC11073124

[5] Xiao C Zhou R Chen Q Hou W Li X Wang Y. Pipeline for assessing tumor immune status using superplex immunostaining and spatial immune interaction analysis. bioRxiv. (2024). doi: 10.1101/2024.08.23.609368

[6] Wattenberg MM Herrera VM Giannone MA Gladney WL Carpenter EL Beatty GL. Systemic inflammation is a determinant of outcomes of CD40 agonist-based therapy in pancreatic cancer patients. JCI Insight. (2021) 6 (5):e145389. doi: 10.1172/jci.insight.145389 33497362 PMC8021099

[7] Balachandran VP Beatty GL Dougan SK. Broadening the impact of immunotherapy to pancreatic cancer: challenges and opportunities. Gastroenterology. (2019) 156:2056–72. doi: 10.1053/j.gastro.2018.12.038 PMC6486864 30660727

[8] Sherman MH Beatty GL. Tumor microenvironment in pancreatic cancer pathogenesis and therapeutic resistance. Annu Rev pathol. (2023) 18:123–48. doi: 10.1146/annurev-pathmechdis-031621-024600 PMC9877114 36130070

[9] Noman MZ Hasmim M Messai Y Terry S Kieda C Janji B. Hypoxia: a key player in antitumor immune response. A Review in the Theme: Cellular Responses to Hypoxia. Am J Physiol Cell Physiol. (2015) 309:C569–79. doi: 10.1152/ajpcell.00207.2015 PMC4628936 26310815

[10] Levental KR Yu H Kass L Lakins JN Egeblad M Erler JT. Matrix crosslinking forces tumor progression by enhancing integrin signaling. Cell. (2009) 139:891–906. doi: 10.1016/j.cell.2009.10.027 19931152 PMC2788004

[11] Orimo A Gupta PB Sgroi DC Arenzana-Seisdedos F Delaunay T Naeem R. Stromal fibroblasts present in invasive human breast carcinomas promote tumor growth and angiogenesis through elevated SDF-1/CXCL12 secretion. Cell. (2005) 121:335–48. doi: 10.1016/j.cell.2005.02.034 15882617

[12] Kalluri R Zeisberg M. Fibroblasts in cancer. Nat Rev Cancer. (2006) 6:392–401. doi: 10.1038/nrc1877 16572188

[13] Piccard H Muschel RJ Opdenakker G. On the dual roles and polarized phenotypes of neutrophils in tumor development and progression. Crit Rev oncology/hematology. (2012) 82:296–309. doi: 10.1016/j.critrevonc.2011.06.004 21798756

[14] Yang Y Cao Y. The impact of VEGF on cancer metastasis and systemic disease. Semin Cancer Biol. (2022) 86:251–61. doi: 10.1016/j.semcancer.2022.03.011 35307547

[15] Goel S Duda DG Xu L Munn LL Boucher Y Fukumura D. Normalization of the vasculature for treatment of cancer and other diseases. Physiol Rev. (2011) 91:1071–121. doi: 10.1152/physrev.00038.2010 PMC3258432 21742796

[16] Mantovani A Marchesi F Malesci A Laghi L Allavena P. Tumour-associated macrophages as treatment targets in oncology. Nat Rev Clin Oncol. (2017) 14:399–416. doi: 10.1038/nrclinonc.2016.217 28117416 PMC5480600

[17] Zhou Z Zhang Y Li J Weng S Li J Chen S. Crosstalk between regulated cell death and immunity in redox dyshomeostasis for pancreatic cancer. Cell signalling. (2023) 109:110774. doi: 10.1016/j.cellsig.2023.110774 37331416

[18] Liu Z Zhou Z Dang Q Xu H Lv J Li H. Immunosuppression in tumor immune microenvironment and its optimization from CAR-T cell therapy. Theranostics. (2022) 12:6273–90. doi: 10.7150/thno.76854 PMC9475465 36168626

[19] Zhou Z Lin T Chen S Zhang G Xu Y Zou H. Omics-based molecular classifications empowering in precision oncology. Cell Oncol (Dordrecht Netherlands). (2024) 47:759–77. doi: 10.1007/s13402-023-00912-8 PMC12974067 38294647

[20] Wang XQ Danenberg E Huang CS Egle D Callari M Bermejo B. Spatial predictors of immunotherapy response in triple-negative breast cancer. Nature. (2023) 621:868–76. doi: 10.1038/s41586-023-06498-3 PMC10533410 37674077

