# Supplementary material for: Case Report: Combined PD-1 and tyrosine kinase blockade stabilizes refractory pancreatic cancer guided by the spatial structure of tumor immune microenvironment
Source: Front Immunol. 2025 May 1;16:1547388. doi: 10.3389/fimmu.2025.1547388 (PMC12078320; doi:10.3389/fimmu.2025.1547388)
Supplement: Supplementary file 2 [file DataSheet2.docx]

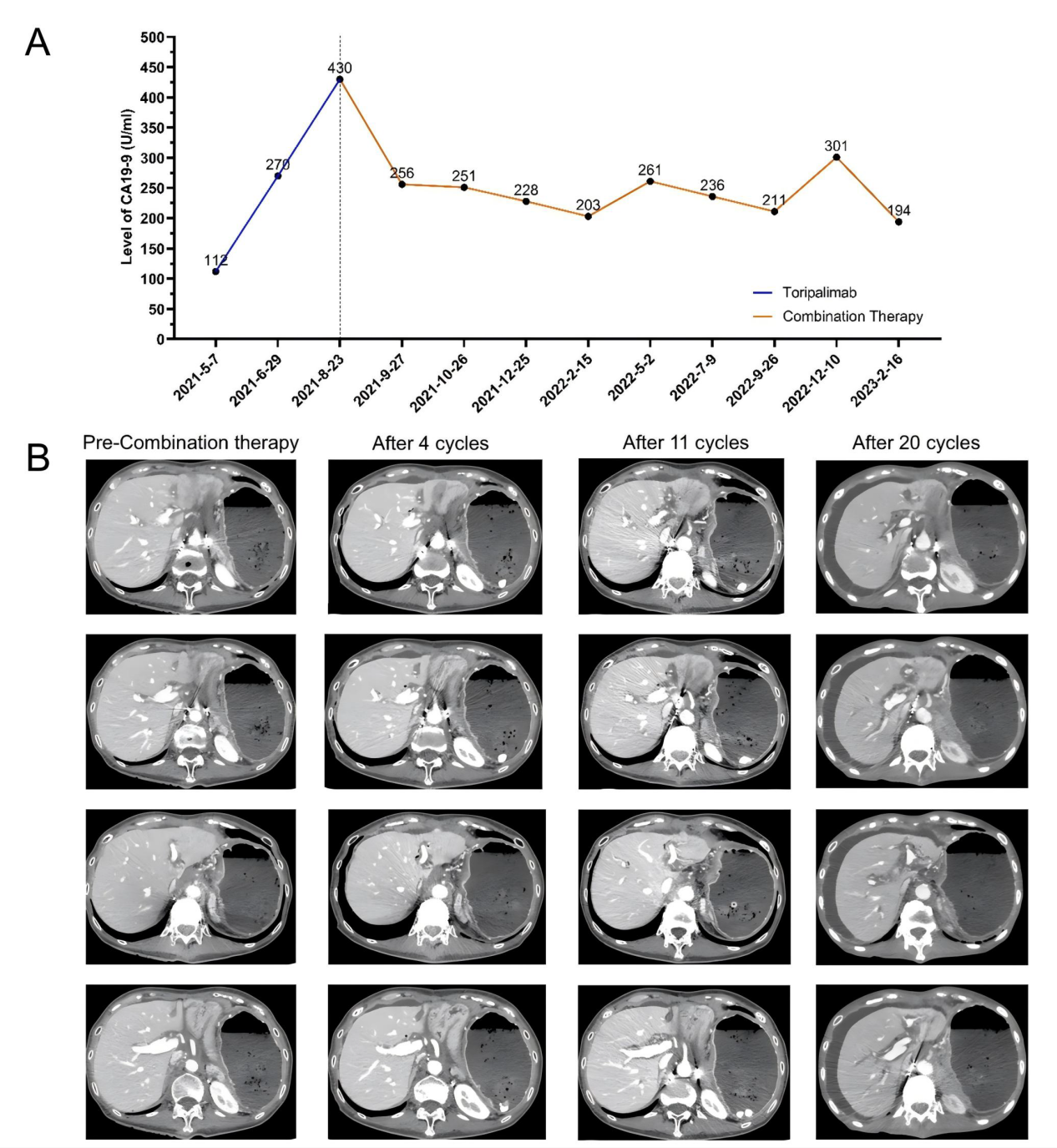


S1. Changes in CA19-9 Levels During Treatment and CT Imaging During Combined Treatment. (A) Dynamic changes in CA19-9 levels over the course of treatment. (B) Comparative CT images of the patient before and after the commencement of combination therapy. The images highlight a stability in tumor size, demonstrating the therapeutic efficacy of the combination treatment.
